# EPID‐based dosimetry to verify IMRT planar dose distribution for the aS1200 EPID and FFF beams

**DOI:** 10.1120/jacmp.v17i6.6336

**Published:** 2016-11-08

**Authors:** Narges Miri, Peter Keller, Benjamin J. Zwan, Peter Greer

**Affiliations:** ^1^ School of Mathematical and Physical Sciences University of Newcastle Callaghan NSW Australia; ^2^ Varian Medical Systems Imaging Laboratory Baden‐Dättwil Switzerland; ^3^ Central Coast Cancer Centre Gosford Hospital Gosford NSW Australia; ^4^ Calvary Mater Newcastle Hospital Newcastle New South Wales Australia

**Keywords:** EPID, dosimetry, IMRT, IMRT treatment plan verification, FFF beams

## Abstract

We proposed to perform a basic dosimetry commissioning on a new imager system, the Varian aS1200 electronic portal imaging device (EPID) and TrueBeam 2.0 linear accelerator for flattened (FF) and flattening filter‐free (FFF) beams, then to develop an image‐based quality assurance (QA) model for verification of the system delivery accuracy for intensity‐modulated radiation therapy (IMRT) treatments. For dosimetry testing, linearity of dose response with MU, imager lag, and effectiveness of backscatter shielding were investigated. Then, an image‐based model was developed to convert images to planar dose onto a virtual water phantom. The model parameters were identified using energy fluence of the Acuros treatment planning system (TPS) and, reference dose profiles and output factors measured at depths of 5, 10, 15, and 20 cm in water phantom for square fields. To validate the model, its calculated dose was compared to measured dose from MapCHECK 2 diode arrays for 36 IMRT fields at 10 cm depth delivered with 6X, 6XFFF, 10X, and 10XFFF energies. An in‐house gamma function was used to compare planar doses pixel‐by‐pixel. Finally, the method was applied to the same IMRT fields to verify their pretreatment delivery dose compared with Eclipse TPS dose. For the EPID commissioning, dose linearity was within 0.4% above 5 MU and ∼1% above 2 MU, measured lag was smaller than the previous EPIDs, and profile symmetry was improved. The model was validated with mean gamma pass rates (standard deviation) of 99.0% (0.4%), 99.5% (0.6%), 99.3% (0.4%), and 98.0% (0.8%) at 3%/3 mm for respectively 6X, 6XFFF, 10X, and 10XFFF beams. Using the same comparison criteria, the beam deliveries were verified with mean pass rates of 100% (0.0%), 99.6% (0.3%), 99.9% (0.1%), and 98.7% (1.4%). Improvements were observed in dosimetric response of the aS1200 imager compared to previous EPID models, and the model was successfully developed for the new system and delivery energies of 6 and 10 MV, FF, and FFF modes.

PACS number(s): 87.53.Oq, 87.53.Xd

## I. INTRODUCTION

Accuracy of dose delivery for IMRT treatments should be determined by an accurate quality assurance (QA) procedure.^(1)^ Recently, there has been a lot of interest in using flattening filter‐free (FFF) beams which give the benefit of reduced headscatter and hence dose outside the field.^(2)^ These beams also deliver the dose faster than flattened beams, which could be beneficial for hypofractionated treatments and reducing intrafractional organ motion.^(3)^ Therefore, they require accurate and efficient quality assurance procedures including patient‐specific quality assurance.

Linear accelerators (linacs) are equipped with EPIDs originally designed for patient positioning,^(4)^ but because EPIDs have high sensitivity, spatial resolution, and immediate digital format, they have also been utilized to determine dose for routine QA of linacs or dose verification of treatments.^5^, ^6^, ^7^, ^8^ The Varian aS1200 EPID detector (Varian Medical Systems, Palo Alto, CA) was released recently and has a large area ($40\times 40\;{\text{cm}}^{2}$), small pixel size (0.0336 cm), and advanced acquisition electronics, and is potentially an improved design for dosimetry.^(9)^ It contains additional backscatter shielding layers to reduce backscatter artifacts from the robotic support arm. It has been adapted by Varian for use in FFF beams without saturation at any source‐to‐detector distance.^10^, ^11^

EPID‐based dosimetry is performed by either (a) simulating the pixel values or (b) converting the pixel values to dose in phantom using a conversion model.^12^, ^13^ The former is based on modeling the detector (EPID) response through Monte Carlo calculation^14^, ^15^ or empirical techniques. The most commonly used empirical model is based on pencil‐beam convolution of a simple fluence model with an EPID dose kernel, and Varian Medical Systems has commercialized this method.^(16)^ For the latter image conversion methods, several mathematical models have been developed to estimate dose to water from EPID images.^17^, ^18^, ^19^, ^20^, ^21^, ^22^ To date, very limited investigations of models to calculate dose in water from EPID images have been reported for high dose‐rate FFF beams, higher energies, and for the new Varian aS1200 EPID design. Podesta et al.^(23)^ reported development of a model for time‐resolved assessment of VMAT for FFF beams for the aS1000 imager; however, they reported time‐dependent gamma evaluations rather than integrated image comparisons.

Recently, an EPID to dose conversion model was developed and validated for 6 MV flattening filter energy (6X), using square field images defined by the multileaf collimator.^(22)^ The model converts images to incident fluence then calculates dose in water using depth‐dependent scatter kernels. They recorded nontransmission images with a prototype backscatter shielded aS1000 EPID and C‐series Varian linac. Gamma comparisons were made to MapCHECK 1 (Sun Nuclear Corporation, Melbourne, FL) measurements for 28 IMRT fields. More recently Keller et al.^(24)^ reported on a Varian implementation of this model for a selection of 6X and 6XFFF fields for the TrueBeam and aS1200 imager comparing converted images to MatriXX (IBA Dosimetry, Schwarzenbruck, Germany) dose measurements.

In this paper, dosimetric testing of the new aS1200 EPID with a Varian TrueBeam linac is performed to verify dose linearity response of the imager, imager lag, and effectiveness/improvement of its backscatter shielding over previous EPID designs. Then, to verify pretreatment dose deliveries, an in‐house image‐to‐dose conversion model is investigated for the EPID using beams at 6 and 10 MV energies and FF and FFF modes. The model parameters are identified using images acquired with open jaw‐defined fields, fluence from the Acuros (Varian), and measured doses in water phantom. To validate the model performance, the modeled dose is compared to planar dose for 36 IMRT fields measured by a MapCHECK 2 detector. Finally, the model was used to verify delivery accuracy in comparison with TPS dose from Eclipse AAA (V.11). This work should allow for efficient and comprehensive verification of conventional and FFF IMRT deliveries for 6 and 10 MV energies.

## II. MATERIALS AND METHODS

### A. Experimental measurements

An aS1200 EPID with a Varian TrueBeam linac (V.2.0) was used to acquire images. The EPID is attached to the gantry through a robotic arm.^(25)^ The active area of the EPID for dosimetry mode is $40\times 40\;{\text{cm}}^{2}$ with 1190×1190 pixel arrays and pixel pitch of 0.336 mm.

To perform the imager dosimetric testing, dose linearity response, lag, and symmetry of the EPID were studied. To verify linearity of the EPID dose response versus delivered dose, $10\times 10\;{\text{cm}}^{2}$ images were acquired at incremental MU irradiations from 2–600 MU, and the central integrated pixel values (IPVs) per MU were plotted against MU. The images were acquired using 6X, 6XFFF, 10X, and 10XFFF beam energies with dose rates of 600, 1400, 600, and 2400 MU/min, respectively. Furthermore, the imager lag or charge carry‐over from frame to frame was examined using frames captured by a frame‐grabber system. The frame‐grabber is a graphic card housed in a separate PC, and connected to the TrueBeam XI node via a unidirectional cable link. The EPID signal was found in a region of interest (ROI) of size $0.33\times 0.33\;{\text{cm}}^{2}$ at the center of each image frame. Finally, to verify the effectiveness of the aS1200 backscatter shielding layers, cross‐plane and in‐plane profiles were compared through the central axis for different size square field images, 2×2, 3×3, 4×4, 6×6, 8×8, 10×10, 15×15, 20×20, and $25\times 25\;{\text{cm}}^{2}$.

Following EPID testing, the model was adapted for pretreatment dose verification for the aS1200 for the higher 10X energy and the FFF modes. To identify the model parameters, for each energy, a set of jaw‐defined square field size (FS) images was acquired, 3×3, 4×4, 6×6, 10×10, 15×15, 20×20, and $25\times 25\;{\text{cm}}^{2}$ at zero gantry angle and 100 cm source‐to‐detector distance (SDD). The Acuros TPS fluence for a $25\times 25\;{\text{cm}}^{2}$ beam was used to identify the parameters of the fluence model. Measured central axis dose and dose profiles of the fields in water phantom were used to identify parameters of the dose model. Dose profiles were measured by an IBA PFD‐3G diode detector and central axis dose was measured by two detectors: a microDiamond (SCD) detector, type 60019 (PTW‐Freiburg GmbH, Freiburg, Germany) with 3.5 mm radius and 45.5 mm length, for $3\times 3\;{\text{cm}}^{2}$ field size and, 0.13 cm^3^ Scanditronix CC13 ion chamber (IBA Dosimetry, Schwarzenbruck, Germany) for the other fields. The measurements were performed in a Scanditronix Wellhofer water tank at depths of 5, 10, 15, and 20 cm, with 100 cm source‐to‐surface distance (SSD).

After parameter identification, to validate the model, the modeling results were compared with measurement results. For validation, integrated EPID images of nine head and neck IMRT fields were acquired at 6X, 6XFFF, 10X, and 10XFFF energies and 100 cm SDD at gantry zero. Delivered dose of each field was recalculated for the same fluence but modified dose rate and energies. This was done to better enable comparison between results for the four energies. These were used to model the dose at 10 cm depth in water. For the same fields, doses were measured with a MapCHECK 2 array (Model 1177, Sun Nuclear Corporation) at 10 cm depth in solid water and 100 cm to the detector plane. An in‐house gamma function was used to compare planar doses pixel‐by‐pixel. The function uses a global dose difference (DD) criteria defined by the percentage of maximum dose of each 2D image plane. All doses above 10% of the maximum dose are assessed with a search region of 6 mm radius.^(26)^ The employed (DD) / (Distance‐to‐Agreement) mm were 3%/3 mm, 2%/2 mm, and 1%/1 mm. All doses are absolute dose as the model converts EPID grayscale images to absolute dose in Gy (i.e., no normalization is performed). The model was then used to verify pretreatment IMRT deliveries by comparison to Eclipse dose planes for the same fields at 10 cm depth using both 3%/3 mm and 2%/2 mm criteria. The IMRT fields were calculated separately on a virtual water phantom with 90 cm SSD and the isocenter at 10 cm depth. Doses were calculated with at 1.5 mm grid size and the three‐dimensional DICOM dose file exported. The TPS dose plane at 10 cm depth was then extracted for comparison to the EPID modelled dose.

### B. Modeling

The method in King et al.^(22)^ was developed to convert EPID images to 2D dose inside a virtual water phantom. This method uses two steps:

Incident fluence modelling
  $$\Psi_{p}(r)=[C_{A}(r)D_{E}(r)]{⊗}^{-1}k_{E}(r)$$
  where $C_{A}(r)$ is a profile correction matrix, $D_{E}(r)$ is EPID image signal matrix, and $k_{E}(r)=e^{-(a_{1}r)}+a_{2}e^{-(a3r)}+a_{4}e^{-(a5r)}$ is the EPID dose deposition kernel.
2. Fluence to dose in water phantom modeling
  $$D_{W}(r)=D_{CAL}.[T(r)\Psi_{p}(r)A(r)]⊗k_{W}(r).\ldots$$
  where $D_{CAL}$ is a calibration factor, $T(r)=1+b_{1}r$ and $A(r)=e^{-{\left(b2r\right)}^{2}}$ are, respectively, terma and attenuation factors, and $k_{W}(r)=b_{3}e^{-{\left(b4r\right)}^{2}}+b_{5}e^{-{\left(b6r\right)}^{2}}+(b_{7}/r)e^{-{\left(b8r\right)}^{2}}$ is the dose deposition in water kernel.

In summary, for the modeling, $C_{A}(r),a_{i}(i=1-5)$, and $b_{j,}(j=1-8)$, require identification. This was done following the procedure outlined in King et al.^(22)^

## III. RESULTS

### A. EPID dosimetry commissioning

**Figure 1:**
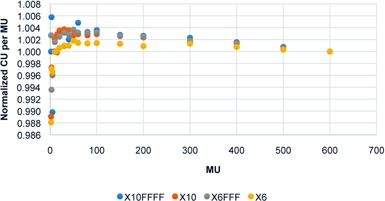
EPID dose response: IPV per MU versus MU (normalized to 600 MU values).

**Figure 2:**
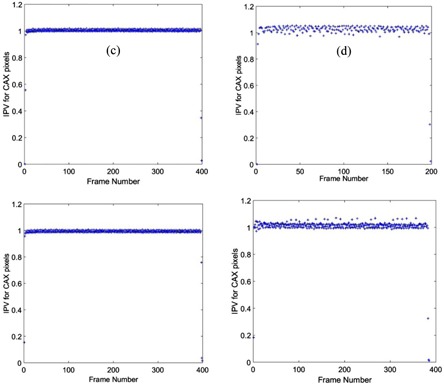
The imager lag for different beam energies. EPID signal in each frame was determined at the central axis, and normalized to the value at frame number 200.

**Figure 3:**
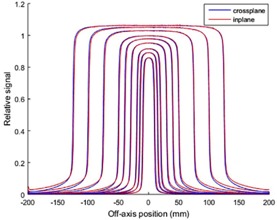
In‐plane/cross‐plane profiles to examine backscatter shielding effectiveness (6X).

Figure 1 demonstrates the EPID dose response linearity. The IPV per MU at central axis was determined for each energy and normalized to the value at 600 MU. Then, the EPID lag was quantified by calculating frame‐by‐frame EPID signal at the central axis. Figure 2 demonstrates the EPID signal versus frame number for the four beam energies. Finally, to examine backscatter shielding effectiveness, cross‐plane and in‐plane profiles were plotted for different square field size images with 6X energy. Figure 3 shows the profiles in both planes.

### B. EPID dose modeling

#### B.1 Fluence profile

**Figure 4:**
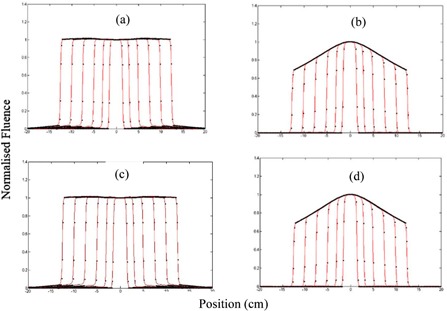
Cross‐plane fluence profile versus field size for different beam energies: (a) 6X, (b) 6XFFF, (c) 10X, and (d) 10XFFF. Model: solid red lines, TPS: black dot lines.

For each beam energy, the EPID kernel parameters were identified using the 25 25 cm^2^ fluence profile from the Acuros and the rest field sizes were used for cross‐validation of the fluence model. The parameters have been summarized in the Appendix, Table A1. Figure 4 demonstrates the agreement between the modeled and the TPS fluence for the field sizes used.

#### B.2 Dose profile

**Figure 5:**
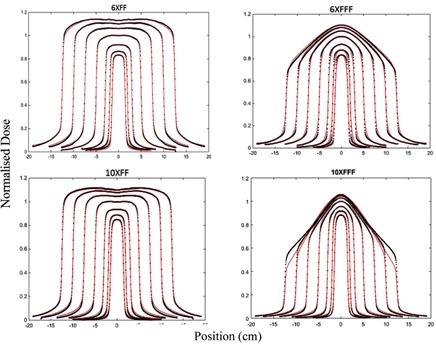
Comparison of modeled and measured cross‐plane dose profiles at 10 cm depth in water. Model: solid red lines, measurements: black dot lines.

**Figure 6:**
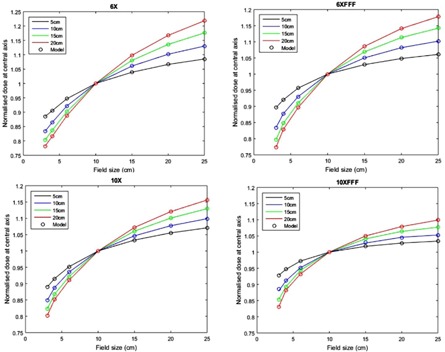
Normalized central axis dose in water versus field size at different depths. Model: circles, measurement: lines.

The parameters of dose calculation in water were identified using measured central axis dose and dose profiles of 3×3, 10×10, 15×15, and $20\times 20\;{\text{cm}}^{2}$ fields at depths of 5, 10, 15, and 20 cm in the water tank and the rest field sizes were used for cross‐validation. The identified parameters are shown in the Appendix, Table A2. For the four beam energies, Fig. 5 illustrates the comparison of the modeled (solid red line) cross‐plane profile from the EPID images and the measured (black dot points) cross‐plane profiles at 10 cm depth in water tank. The figure includes both training and cross‐validation results. All dose profiles were normalized to the central axis dose of the 10x10 cm^2^ image. Figure 6 demonstrates comparison of the modeled and measured central axis dose for all beam energies at the four different depths in water. All doses have been normalized to the 10x10 cm^2^ field dose.

#### B.3 Model validation

To validate the model performance, the dose for nine IMRT head and neck fields were modeled from EPID images and compared to the measured doses with MapCHECK 2. The validation results have been summarized in Table 1.

**Table 1:** Model validation using MapCHECK 2 measurements

	*6X*			*6XFFF*		
*Fields*	*3%/3mm*	*2%/2mm*	*1%/1mm*	*3%/3mm*	*2%/2mm*	*1%/1mm*
1	99.6	95.9	76.6	100.0	99.0	80.6
2	99.1	87.2	61.0	98.1	92.4	59.7
3	99.3	92.9	68.0	99.9	97.1	70.3
4	98.9	92.7	67.6	99.8	97.3	71.2
5	98.9	94.2	66.1	99.6	91.8	62.0
6	98.9	94.2	66.1	99.9	98.2	77.8
7	99.0	95.7	75.3	99.9	98.5	82.5
8	98.3	90.5	56.9	99.0	91.6	65.3
9	99.4	93.9	71.6	99.1	95.3	61.6
Mean (SD)	99.0 (0.4)	93.0 (2.7)	67.7 (6.3)	99.5 (0.63)	95.7 (3.0)	70.0 (8.6)

	*10X*			*10XFFF*		
*Fields*	*3%/3mm*	*2%/2mm*	*1%/1mm*	*3%/3mm*	*2%/2mm*	*1%/1mm*
1	99.7	96.2	75.7	99.2	96.0	74.3
2	99.6	93.2	67.9	97.0	85.6	57.3
3	99.3	93.6	71.9	97.8	90.9	63.0
4	98.9	93.9	73.0	98.4	89.0	63.4
5	99.6	95.0	73.0	98.0	89.6	64.4
6	99.5	94.8	71.5	98.3	98.1	92.9
7	99.0	95.4	72.8	98.9	93.8	79.4
8	98.7	93.8	70.3	97.4	89.9	57.8
9	99.8	95.3	77.4	96.7	88.2	58.4
Mean (SD)	99.3 (0.39)	94.6 (0.99)	72.6 (2.8)	98.0 (0.84)	91.2 (4.0)	67.9 (12.0)

#### B.4 Model performance

**Figure 7:**
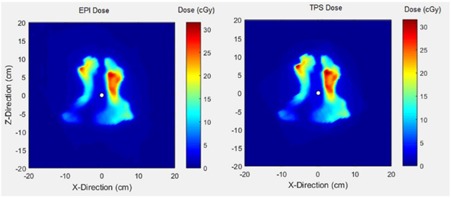
Dose matrix for a head and neck field of a 6XFFF beam with the modeled dose (left‐side) and TPS dose (right‐side) at 10 cm depth in water.

Finally, the modeled dose was compared to the TPS dose for the same fields. The comparison results have been summarized in Table 2. Figure 7 shows an example of the model performance compared with the TPS dose.

**Table 2:** Pretreatment verification using the model compared to TPS dose at 10 cm depth

	*6X*			*6XFFF*		
*Fields*	*3%/3mm*	*2%/2mm*	*1%/1mm*	*3%/3mm*	*2%/2mm*	*1%/1mm*
1	100.0	99.8	81.6	100.0	98.6	74.2
2	100.0	96.4	66.0	99.5	90.6	50.9
3	99.9	96.7	81.8	99.8	97.1	71.2
4	100.0	99.9	88.0	99.1	97.3	68.9
5	100.0	99.9	88.7	99.4	90.3	57.9
6	100.0	99.9	88.7	99.9	98.0	72.7
7	100.0	99.3	89.0	100.0	99.2	78.7
8	100.0	99.8	84.0	99.3	94.4	67.3
9	100.0	100.0	93.9	99.8	93.9	61.2
Mean (SD)	100.0 (0.0)	99.1 (1.4)	84.6 (8.0)	99.6 (0.33)	95.5 (3.4)	67.0 (8.8)

	*10X*			*10XFFF*		
*Fields*	*3%/3mm*	*2%/2mm*	*1%/1mm*	*3%/3mm*	*2%/2mm*	*1%/1mm*
1	100.0	99.8	78.3	99.9	99.2	83.1
2	99.8	98.5	71.7	98.2	88.1	58.5
3	100.0	97.0	79.7	98.2	94.1	68.8
4	100.0	99.7	84.5	97.9	91.9	68.5
5	100.0	99.1	85.6	98.6	92.6	68.4
6	100.0	99.6	78.9	99.9	99.7	98.1
7	100.0	99.2	81.7	99.9	99.0	80.5
8	99.8	98.6	83.0	98.9	94.4	65.3
9	99.9	99.6	88.1	96.7	89.0	61.7
Mean (SD)	99.9 (0.09)	99.0 (0.89)	81.3 (4.8)	98.7 (1.1)	94.2 (4.3)	72.5 (12.5)

## IV. DISCUSSION

Initially, this paper outlines major dosimetry tests performed to commission the new aS1200 EPID system on the TrueBeam accelerator. The linearity of the EPID dose response was within 0.4% above 5 MU and ∼1% above 2 MU. This linearity of response is a considerable improvement over previous reports for both Varian IAS3 and other vendor EPID systems which show under‐response of 3%–5% for small MU.^27^, ^28^, ^29^ Moreover, the measured lag for the EPID was found to be extremely small compared with previous reports, which had shown lag effects of several percent with signal increasing with increasing MU due to charge carry‐over. No increase in image signal with MU was apparent for the aS1200 EPID model. Furthermore, the symmetry of the profiles for the EPID was considerably improved over the aS1000 imager, indicating the effectiveness of the backscatter shielding in the new system.^29^, ^30^, ^31^ This was previously investigated with a prototype shielded panel.^(22)^ Studies on aS1000 imagers have demonstrated around 8% additional nonuniform backscatter to the panel introduces dosimetry artifacts.^(30,32–34)^ Combined with the active repositioning of the detector specified to within 0.5 mm for all gantry angles, these results suggest that the aS1200 has excellent properties for dosimetry and is clearly superior to previous models.

Secondly, to verify delivery dose, a kernel‐based model was employed to determine delivered dose to a virtual flat water phantom. The model input is images acquired with EPIDs and its output is dose onto the virtual phantom. Jaw‐defined fields were used to identify the model parameters for aS1200 imager; however, in King et al.^(22)^ MLC‐defined fields were used. While MLC‐defined fields should accurately account for the phantom scatter, they do not incorporate the variation in dose due to headscatter, which then may require a separate correction factor. To identify the model parameters, TPS fluence and measured dose from square field irradiations inside a rectangular water phantom were utilized. As Figs. 5 and 6 illustrate, modeled dose profiles closely follow the measured profiles for square field irradiations. Disagreement between the modeled and measured results was slightly larger for 6XFFF profiles and large field sizes of 10XFFF profiles. This could be because the model was originally developed to model flattening filter beams. The reduced performance of the model for FFF beams is likely due to the more complex structure of FFF beam profiles with field size. This structure also makes kernel parameter identification more difficult. Adaptations to the model to improve this could include an improved off‐axis model for FFF deliveries. Another possibility would be to investigate whether the 6X and 10X kernels can accurately model the FFF beams allowing parameter identification to concentrate on modelling the beam profiles for these beams. The model comparison to measured MapCHECK data gave average gamma values over 99% for three energies and 98% for 10XFFF. These were assessed only at 3%/3 mm criteria as the MapCHECK is a low‐resolution dosimeter with detector spacing of 7.07 mm.

To ultimately validate the model for clinical fields, modeled dose was compared with measured dose. Table 1 shows the validation results at three gamma criteria. According to this table, for all four energies, the modeled dose had more than 97% agreement with measured dose at 3%/3 mm criteria. Using tighter criteria, the lowest mean pass rates were 91.2% and 67.7% respectively for 2%/2 mm and 1%/1 mm criteria. This relatively poor accuracy for the more stringent criteria could come from MLC interleaf leakage alignment with diode detectors in MapCHECK, detector limitation in measurement, and/or human errors. Altogether, the validation results show a slight improvement over similar studies comparing their model with MapCHECK measurements.^(35)^ Finally, the model was used to verify pretreatment deliveries of the same clinical fields in comparison with corresponding TPS prescribed dose. According to Table 2, more than 99% and 94% pixel similarity was observed at respectively 3%/3 mm and 2%/2 mm. However, one may observe the higher pass rates when comparing to TPS than the MapCHECK measurements, similar to other studies.^35^, ^36^ This is possibly due to smaller number of detectors in MapCHECK compared to the EPID and measurement uncertainties.

## V. CONCLUSIONS

Images from electronic portal imaging device (EPID) provide an efficient tool to verify pre‐treatment delivery dose for radiation therapy. In this paper, a model was derived to estimate the dose inside a virtual flat water phantom for the aS1200 EPID and flattened and FFF beams at 6 and 10 MV. The model parameters were identified using measured dose in water phantom for open field beams. Then, the model performance for IMRT planar fields was validated in comparison with MapCHECK measurements at 10 cm depth in solid water. The model later verified delivery dose of 36 IMRT fields.

## ACKNOWLEDGMENTS

Funding has been provided from the Department of Radiation Oncology, TROG Cancer Research, and the University of Newcastle. Narges Miri is a recipient of a University of Newcastle postgraduate scholarship.

## COPYRIGHT

This work is licensed under a Creative Commons Attribution 3.0 Unported License.

## APPENDICES

### Appendix A. Identified parameters for kernels

**Table A1:** Identified parameters of the EPID kernel for different beam energies

*Profile*	$a_{1}$	$a_{2}$	$a_{3}$	$a_{4}$	$a_{5}$
6X	34.99996	0.00025	1.28729	$0.20810\times 10^{-6}$	0.00010
6XFFF	34.99910	0.00040	1.80128	$0.10621\times 10^{-4}$	0.32360
10X	34.99999	0.00024	1.12503	$0.36956\times 10^{-6}$	0.00010
10XFFF	34.99910	0.00042	1.91661	$0.13382\times 10^{-4}$	0.31100

**Table A2:** Identified parameters of the dose kernel for different beam energies

	$b_{1}$	$b_{2}$	$b_{3}$	$b_{4}$	$b_{5}$	$b_{6}$	$b_{7}$	$b_{8}$
*6X*								
5 cm	0.00518	0.00037	0.99515	5.39435	0.00339	1.22809	0.00146	0.06500
10 cm	0.00553	0.00057	0.99281	5.61374	0.00323	1.07309	0.00387	0.07789
15 cm	0.00555	0.00077	0.98969	5.51726	0.00249	1.02052	0.00782	0.09486
20 cm	0.00479	0.00081	0.98745	5.79484	0.00189	1.10226	0.01066	0.10108
*6FFF*								
5 cm	0.00297	$4.233\times 10^{-10}$	0.99456	7.80186	$1.18\times 10^{-10}$	0.41998	0.00544	0.18896z
10 cm	0.00381	$1.048\times 10^{-10}$	0.96347	14.9991	0.03170	3.31156	0.00483	0.16257
15 cm	0.00310	$2.341\times 10^{-9}$	0.95928	14.9991	0.03424	3.23009	0.00648	0.14223
0 cm	0.00206	$2.287\times 10^{-9}$	0.99287	9.91172	$5.92\times 10^{-10}$	0.18822	0.00707	0.18876
*10X*								
5 cm	0.00312	0.00013	0.99451	8.01828	0.00536	1.56025	0.00012	$1.13\times 10^{-6}$
10 cm	0.00285	0.00029	0.99470	8.71554	0.00479	1.39946	0.00051	0.02454
15 cm	0.00196	0.00031	0.99497	10.5116	0.00396	1.44461	0.00107	0.06937
20 cm	0.00210	0.00049	0.99658	14.9910	0.00245	1.53943	0.00097	0.09055
*10XFFF*								
5 cm	$2.880\times 10^{-9}$	0.00328	0.98490	6.96354	0.00019	0.28823	0.01502	0.99999
10 cm	$4.869\times 10^{-9}$	0.003377	0.990033	6.414833	$1.41\times 10^{-11}$	0.471982	0.009967	0.171306
15 cm	$4.42\times 10^{-10}$	0.003379	0.987812	14.99998	$7.853\times 10^{-5}$	0.248740	0.012109	0.999985
20 cm	$1.015\times 10^{-8}$	0.003579	0.989572	7.275969	$1.08\times 10^{-11}$	0.768778	0.010428	0.137472

## Supporting information

Supplementary Material
