# Supplementary material for: EPID‐based dosimetry to verify IMRT planar dose distribution for the aS1200 EPID and FFF beams
Source: J Appl Clin Med Phys. 2016 Nov 8;17(6):292–304. doi: 10.1120/jacmp.v17i6.6336 (PMC5690494; doi:10.1120/jacmp.v17i6.6336)
Supplement: Supplementary file 1 — Supplementary Material [file ACM2-17-292-s001.doc]

| Section | Feedback | Author Respond |
| --- | --- | --- |
| **Introduction** | Line 46 – “and these require rigorous verification.”  This statement should be changed to something more accurate and more reflective of the true motivation of your paper.  It has not been demonstrated that FFF IMRT fields demand any more rigorous verification than any other IMRT field. | Below sentences were added:  ‘Recently, there has been a lot of interest in using flattening filter free (FFF) beams which give the benefit of reduced head-scatter and hence dose outside the field . These beams also deliver the dose faster than flattened beams which could be beneficial for hypo-fractionated treatments and reducing intrafractional organ motion . Therefore, they require accurate and efficient quality assurance procedures including patient-specific quality assurance. ‘ |
| **Introduction** | Line 61 “have” à “has” | It was changed to  ‘has’ |
| **Introduction** | Line 75  remove “limited” | ‘Limited’ was removed. |
| **Introduction** | Lines 80-81 the last two sections of this sentence are messy.  Re-write. | The sentences were modified to  ‘In this paper, dosimetric testing of the new aS1200 EPID with a Varian TrueBeam linac is performed to verify dose linearity response of the imager, imager lag and effectiveness/improvement of its backscatter shielding over previous EPID designs.’ |
| **Experimental Measurements** | Line 95 reconcile imager size with Line 51. | In the second statement, 40x40 is the active area, however, I reconciled them. |
| **Experimental Measurements** | Line 119 missing dimensions | The dimensions were added as:  ‘Dose profiles were measured by an IBA PFD-3G diode detector and central axis dose was measured by two detectors: a MicroDiamond (SCD) detector, type 60019 with 3.5 mm radius and 45.5 mm length, for 3×3 cm^2^ field size and, 0.13 cm^3^ Scanditronix CC13 ion chamber for the other fields.’ |
| **Experimental Measurements** | Lines 125-126 incomplete sentence | The sentence was modified to:  ‘For validation, integrated EPID images of nine head and neck IMRT fields were acquired at 6X, 6XFFF, 10X and 10XFFF energies and 100 cm SDD at gantry zero. Delivered dose of each field was recalculated for the same fluence but modified dose rate and energies.’ |
| **Modelling** | Line 153 “in King et al.” should be “in Reference (30)” or similar | The author names were removed and the reference number was substituted. |
| **Discussion** | Lines 196-198 “Previous work has shown…” is a poorly worded sentence which needs reconstruction. | The sentence was reworded as:  ‘Studies on aS1000 imagers have demonstrated around 8% additional non-uniform backscatter to the panel introduces dosimetry artefacts .’ |
| **Discussion** | Lines 203-206 “Jaw defined fields…” is a confusing sentence, and difficult to reconcile with your response to my previous review. Please clarify. | The sentences were clarified as:  ‘Jaw defined fields were used to identify the model parameters for aS1200 imager however, in MLC defined fields were used. While MLC defined fields should accurately account for the phantom scatter, they do not incorporate the variation in dose due to head scatter which then may require a separate correction factor.’ |
| **Discussion** | Lines 209-210 “Deviations are…” Reword for clarity | The sentence was reworded as:  ‘Disagreement between the modelled and measured results was slightly larger for 6XFFF profiles and large field sizes of 10XFFF profiles. This could be because the model was originally developed to model flattening filter beams. The reduced performance of the model for FFF beams is likely due to the more complex structure of FFF beam profiles with field size.’ |
| **Discussion** | Line 212 “likely due” not “likely to be due” | The sentence was modified to:  ‘The reduced performance of the model for FFF beams is likely due to the more complex structure of FFF beam profiles with field size.’ |
| **Discussion** | Lines 221-229 This whole paragraph is structured and worded very poorly.  Please revise for clarity. | The whole paragraph of Discussion section was modified to:  ‘To ultimately validate the model for clinical fields, modelled dose was compared with measured dose. Error: Reference source not found shows the validation results at three gamma criteria. According to this table, for all four energies, the modelled dose had more than 97% agreement with measured dose at 3%/3 mm criteria. Using tighter criteria, the lowest mean pass rates were 91.2% and 67.7% respectively for 2%/2 mm and 1%/1 mm criteria . This relatively poor accuracy for the more stringent criteria could come from MLC interleaf leakage alignment with diode detectors in MapCheck, detector limitation in measurement and/or human errors. Altogether, the validation results show a slight improvement over similar studies comparing their model with MapCheck measurements . Finally, the model was used to verify pre-treatment deliveries of the same clinical fields in comparison with corresponding TPS prescribed dose. According to Error: Reference source not found, more than 99% and 94% pixel similarity was observed at respectively 3%/3 mm and 2%/2 mm. However, one may observe the higher pass rates when comparing to TPS than the MapCheck measurements, similar to other studies . This possibly due to smaller number of detectors in MapCheck compared to the EPID and measurement uncertainties.  ‘ |
| **Discussion** | I do not know what you mean by global dose criteria (which you never define, and seem to confuse with the 10% dose threshold twice elsewhere) “is however inherently more sensitive to dose discrepancies in higher dose regions.”  Explain. | Explanation was added to line 145 in ‘Experimental measurements’ section.  ‘An in-house gamma function was used to compare planar doses pixel-by-pixel. The function uses a global dose difference (DD) criteria defined by the percentage of maximum dose of each 2D image plane. All doses above 10% of the maximum dose are assessed with a search region of 6 mm radius . The employed (DD)/ (Distance-to-Agreement) mm were 3%/3 mm, 2%/ 2 mm and 1%/1 mm. All doses are absolute dose as the model converts EPID grayscale images to absolute dose in Gy, i.e. no normalization is performed. |
| **Major comment** | How did you perform your gamma analyses?  I did not see any indication in the manuscript concerning whether you used an in-house algorithm, or performed the analysis in SNC Patient or Eclipse.   Gamma analysis results should never be quoted without a full description of how the calculation was performed.  What was your region of interest or dose threshold?  Did you perform local or global analysis?  Were you analyzing absolute dose or relative dose? | Gamma analysis was not by Eclipse/SNC but a local gamma function. Added to the last paragraph in ‘Experimental measurements’ section. See above response for text. |
| **Major comment** | It is not true that simply because you used open-field measurements to commission your algorithm that it is then unnecessary to subsequently measure and analyze open fields (or very simply MLC fluences) with the completed algorithm. | The model parameters were identified using open field data then some cross-validation was performed for open field images (we called this stage as parameter identification stage). To not make the paper confusing, both training and validation results are taken in the Figure 5. Then, the model was validated for clinical fields comparing with measurements.  For clarity, Dose profile paragraph in Result section was modified to:  ‘The parameters of dose calculation in water were identified using measured central axis dose and dose profiles of 3×3, 10×10, 15×15 and 20×20 cm^2^ fields at depths of 5, 10, 15 and 20 cm in the water tank and the rest field sizes were used for cross validation. ‘ |
| **Major comment** | Secondly, the fact that the SNC Patient software interpolates the MapCHECK 2 and EPID data to a 1 mm2 grid does not exonerate you of validating your algorithm with appropriately rigorous gamma criteria. | The measurements were performed by MapCheck2 and an in-house gamma function was used for all comparisons. The gamma function description was added to the last paragraph in ‘Experimental measurements’  See above response for text. |
| **Major comment** | Without the addition of the 2%, 2 mm data included now in Table 2, your results presentation would be total unacceptable.  1%, 1 mm is most definitely “achievable.” | The results for tighter gamma (2%/2 mm and 1%/1 mm) were added to tables 1&2 and some similar literature was added to the last paragraph of Discussion section. |
| **Major comment** | Lastly, your comment that “there are no currently accepted methods to analyse these type of data…”, in the context of the location within the field of gamma failures, is not correct: linear dose profiles are consistently used clinically and published to show measurement accuracy in areas of high dose gradient, penumbra regions, dose outside the field, etc. | We added the 1%, 1 mm gamma results that use the interpolation. |

 
